# Women’s autonomy and maternal healthcare service utilization in Ethiopia

**DOI:** 10.1186/s12913-017-2670-9

**Published:** 2017-11-13

**Authors:** Fentanesh Nibret Tiruneh, Kun-Yang Chuang, Ying-Chih Chuang

**Affiliations:** School of Public Health, Taipei Medical University, 250 Wu-Hsing Street, Taipei City, Taiwan

**Keywords:** Antenatal care, Health facility delivery, Postnatal care, Maternal healthcare utilization, Community-level, Individual-level, Ethiopia

## Abstract

**Background:**

Most previous studies on healthcare service utilization in low-income countries have not used a multilevel study design to address the importance of community-level women’s autonomy. We assessed whether women’s autonomy, measured at both individual and community levels, is associated with maternal healthcare service utilization in Ethiopia.

**Methods:**

We analyzed data from the 2005 and 2011 Ethiopia Demographic and Health Surveys (*N* = 6058 and 7043, respectively) for measuring women’s decision-making power and permissive gender norms associated with wife beating. We used Spearman’s correlation and the chi-squared test for bivariate analyses and constructed generalized estimating equation logistic regression models to analyze the associations between women’s autonomy indicators and maternal healthcare service utilization with control for other socioeconomic characteristics.

**Results:**

Our multivariate analysis showed that women living in communities with a higher percentage of opposing attitudes toward wife beating were more likely to use all three types of maternal healthcare services in 2011 (adjusted odds ratios = 1.21, 1.23, and 1.18 for four or more antenatal care visits, health facility delivery, and postnatal care visits, respectively). In 2005, the adjusted odds ratios were 1.16 and 1.17 for four or more antenatal care visits and health facility delivery, respectively. In 2011, the percentage of women in the community with high decision-making power was positively associated with the likelihood of four or more antenatal care visits (adjusted odds ratio = 1.14). The association of individual-level autonomy on maternal healthcare service utilization was less profound after we controlled for other individual-level and community-level characteristics.

**Conclusions:**

Our study shows that women’s autonomy was positively associated with maternal healthcare service utilization in Ethiopia. We suggest addressing woman empowerment in national policies and programs would be the optimal solution.

**Electronic supplementary material:**

The online version of this article (10.1186/s12913-017-2670-9) contains supplementary material, which is available to authorized users.

## Background

Although most maternal deaths are preventable, the most recent available data indicate that the maternal mortality rate in Africa is among the highest worldwide [1]. According to the 2015 World Health Organization (WHO) report, 303,000 women worldwide died during pregnancy or after childbirth, and most of these deaths occurred in sub-Saharan Africa [2]. In Ethiopia (the current study site), the maternal mortality ratio is considerably high—353 per 100,000 live births [3]. Although significantly reducing maternal mortality in sub-Saharan African countries appears difficult, skilled care before, during, and after childbirth is highly crucial for saving the lives of women and newborn infants [4–6]. Maternal healthcare service utilization is strongly recommended as the most critical strategy for reducing maternal mortality rates in low-income countries [7, 8].

Studies have shown that in Ethiopia, maternal healthcare service utilization is below acceptable standards [9]. Only 19% of women made four or more antenatal care (ANC) visits during their pregnancy, only 11% of pregnant women had health facility delivery (HFD), and only 9.7% of women received postnatal care (PNC) in the first 2 days after delivery [10]. Reasons for the low maternal healthcare service utilization include the lack of transportation, a long distance to a health facility, cultural beliefs inculcating a preference for home births, fear of health facility treatments, and lack of awareness regarding the importance of skilled delivery [11].

Most of the aforementioned barriers are related to community norms toward maternal healthcare services and women’s autonomy, both of which have received little attention previously [12]. Women’s autonomy is a multidimensional concept, including both control over resources (physical, human, intellectual, and financial) and ideologies (beliefs, values, attitudes, internal strength, self-esteem, and self-confidence) [13]; this entails increased self-confidence and inner transformation of one’s consciousness, which enable the woman to overcome external barriers and access resources or change traditional ideologies [14]. To measure women’s autonomy, previous studies have used two key aspects—decision-making ability and attitudes toward domestic violence [15]. Women with high autonomy are assumed to be more likely to have high self-esteem, to not accept gender inequalities in power, and to disagree with any justification for wife beating. Many studies have shown decision-making ability and attitudes toward domestic violence to be valid measures for assessing women’s autonomy [16–18].

Studies have demonstrated that both individual-level and community-level autonomies can influence a woman’s decision to seek care [19, 20]. At the individual level, low autonomy can affect women’s health through fewer opportunities to engage in paid employment, presence of domestic violence, and limited access to healthcare services [21, 22]. Studies measuring women’s autonomy at the individual level have revealed that women who can make their own decisions regarding medical care and oppose attitudes toward domestic violence are more likely to receive professional ANC and HFD [23–27]. This relationship remains significant even after controlling for several socioeconomic and demographic factors.

At the community level, low autonomy can affect women’s health through cultural beliefs and practices. Most sub-Saharan African cultures consider women leaving their homes to seek healthcare unacceptable [28]. In particular, a study in rural sub-Saharan Africa showed that women living in communities where women are expected not to visit healthcare facilities alone are less likely to use ANC and skilled delivery [19]. By contrast, if the community norms support women’s own decision-making in healthcare seeking, women are more likely to use various healthcare services [25]. For instance, a study in Colombia suggested that women’s autonomy at the community level was one of the greatest contributing factors for completing four or more ANC visits [29]. Similarly, studies have shown that women living in an area with norms supporting gender equality, measured through attitudes against wife beating, were more likely to deliver with the assistance of a health professional, to have four or more antenatal visits, or to start their prenatal visits in the first trimester than those living in areas with a high tolerance of wife beating were [19].

Studies on the relationship between women’s autonomy and healthcare service utilization have had several limitations. First, most studies in Ethiopia, focused only on individual-level and household-level characteristics and did not examine the community determinants of healthcare service utilization. Second, the previous studies in this country mainly focused on particular regions or districts, limiting their generalizability [12, 30, 31]. Therefore, in this study, we included both individual-level and community-level indicators to assess whether women’s autonomy is associated with maternal healthcare service utilization in Ethiopia. We hypothesized that a higher level of women’s autonomy and socio-economic status at both the individual and community levels is associated with higher utilization of maternal healthcare service.

## Methods

### Data

This study used data from the 2005 and 2011 Ethiopia Demographic and Health Surveys (EDHS). Stratified two-stage cluster sampling was used to select households for the survey. The sampling method was used to randomly select clusters (communities) in the first stage, households in the second stage, and household members in the final stage. Both the 2005 and 2011 EDHS defined a cluster as a census enumeration area containing 150–200 households [32].

The 2005 EDHS sample contained 14,500 households from 535 clusters, and 14,070 women aged 15–49 years were interviewed; the response rate was 99%. The 2011 EDHS sample comprised 17,817 households, and 16,515 women aged 15–49 were interviewed; the response rate was 95%. The number of clusters was 596. The average number of women interviewed per cluster was 26 and 28 for the 2005 and 2011 surveys, respectively.

In both surveys, face-to-face structured interviews were conducted. Our analysis focused on married women or women living with a male partner of reproductive age (15–49 years) who had a live birth within 5 years before the survey (6058 and 7043 women in the 2005 and 2011 EDHS, respectively). If women had more than one birth in the previous 5 years, we analyzed only the most recent birth to minimize the recall bias and to avoid the clustering effects of multiple singleton births to the same mother. The sampling procedure is described in detail in a report on the EDHS sampling design [10]. Because the number of missing values is relatively small, we excluded these missing cases from the analyses.

### Measures

#### Outcome variables

The utilizations of ANC, HFD, and PNC for the most recent live birth within 5 years before the surveys were selected as the outcome measures. For ANC, we used a dichotomous variable to indicate whether a woman attended four or more ANC visits with a trained healthcare provider (0 = no, 1 = yes). For HFD, we noted whether a woman delivered her baby at a health facility (0 = no, 1 = yes). In Ethiopia, if childbirth occurs at home, it is unlikely that the birth delivery is assisted by a trained healthcare provider [32]. For PNC, we noted whether a woman received any PNC service from a health professional or a trained healthcare provider within 42 days after delivery (0 = no, 1 = yes) [33].

#### Individual-level variables

We measured two aspects of women’s autonomy: decision-making power in the household and attitudes toward wife beating [15]. Decision-making power in the household was measured using the answers to the following three questions: who decides matters pertaining to (a) the woman’s health (personal decision-making authority), (b) major purchases (economic decision-making authority), and (c) visits to friends or family (mobility decision-making authority)? A woman who made more than one decision, either alone or jointly with her husband, was categorized as having high decision-making authority [27]. A woman who made one or no decision was categorized as having low decision-making authority. Attitudes toward wife beating or domestic violence were measured on the basis of five hypothetical scenarios: (1) she goes out without telling him, (2) she neglects the children, (3) she argues with him, (4) she refuses to have sexual intercourse with him, and (5) she does not cook food properly. If the respondent agreed that her husband had a right to beat her in any of these five hypothetical scenarios, she was classified as having favorable attitudes toward domestic violence against women. If she did not agree with any of these hypothetical scenarios, she was classified as having opposing attitudes toward domestic violence against women.

We also included a number of socioeconomic and demographic factors in our analyses because they are key to understand maternal healthcare service utilization [34]. We included mother’s age (15–24, 25–34, or 35–49 years), age at first marriage (<16, 16–19, or ≥20 years), religion (Orthodox Christianity, Protestant Christianity, Islam, or others), place of residence (urban or rural) number of children (1–2, 3–4, or ≥5) and whether the child was wanted at the time of pregnancy (yes or no), educational level (none, primary, secondary, and higher), the spouse’s educational level (none, primary, secondary, or higher), employment status (no or yes), wealth index (poorest, poor, middle, rich, and richest). The wealth index was a composite score measured according to household assets such as televisions, bicycles, materials used for house construction, types of water access, sanitation facilities, and other wealth-related characteristics. Factor scores of household assets were generated through a principal component analysis and were then standardized and categorized into five quintiles—poorest, poor, middle, rich, and richest [10, 35].

Another factor included in our analysis is media exposure (no or yes). A large percentage of population in low- and middle-income countries considered mass media is the main source of health and health seeking information [36]. Therefore, our study assessed whether the respondents had been exposure to any of the three types of mass media, namely radio, television, and newspapers or magazine.

#### Community-level variables

We included five continuous community-level variables in our study, which were obtained by aggregating individual responses for each item to the community (cluster) level. Specifically, before our analyses, the variables were coded as 0 and 1. Then we calculated the percentages within a given category for each community-level variable. The five community-level variables were the proportions of women who received primary and higher education, were employed, had negative attitudes toward wife beating, had high decision-making power, and used contraception. These variables represented the community norms and social contexts regarding women’s autonomy, the availability of healthcare services, and the socioeconomic status of the community [35].

#### Statistical analyses

All analyses were conducted using Statistical Analysis Software (SAS) (version 9.4). Data from the 2005 and 2011 EDHS were separately analyzed to examine changes in maternal healthcare service utilization and women’s autonomy between the two periods; these results could indicate progression of maternal healthcare service utilization and changes in the relevant factors. We conducted a series of bivariate and multivariate analyses. We used a chi-squared test to examine the association of individual-level characteristics with maternal healthcare service utilization. Because of our community-level variables being nonnormally distributed, we used Spearman’s correlation to analyze associations among community-level characteristics. For the multivariate analysis, we constructed a series of generalized estimating equation logistic regression models to examine the relationships of explanatory variables with outcomes, while controlling for confounding factors. Because individuals living in the same community may be more similar to each other than individuals from different communities, observations of individuals within a community are not necessarily independent. Generalized estimating equation models were used to adjust the correlated individual responses within a single community. Results of the multivariate relationships were expressed as adjusted odds ratios (AORs) with 95% confidence intervals (95% CIs). We first constructed a null model (Model 1). We then included only individual-level variables in Model 2 and only community-level variables in Model 3. Finally, we simultaneously included both individual-level and community-level variables in Model 4 (final model). We present only the results of the final model.

We calculated intraclass correlation coefficients (ICCs) for the multivariate regression models, which represent the proportion of variance at the group level divided by the sum of the variances at the individual level and the group level. According to Snijders and Bosker (2012), for a binary outcome, the unobserved individual variance follows a logistic distribution with individual level variance V_i_ equal to π^2^/3 (i.e., 3.29) [37]. Therefore, the ICC for binary outcomes is calculated with V_A_ representing variance between groups (communities). We examined multicollinearity problems in the final regression models including all individual-level and community-level characteristics by estimating the variance inflation factor and tolerance. All tolerance values were >0.1, and all variance inflation factor values were <10. Therefore, no multicollinearity problems were observed in the independent variables in the regression models.

#### Ethical considerations

Data collection procedures for the EDHS were approved by the Ethical Review Board of the Ethiopia Health and Nutrition Research Institute, the National Research Ethics Review Committee of the Ministry of Science and Technology, the Institutional Review Board of International Classification of Functioning, and the Centers for Disease Control and Prevention. Verbal informed consent was obtained from each respondent owing to limited literacy levels in the population [10]. We obtained approval to use the data from the DHS repository (http://dhsprogram.com/data/available-datasets.cfm).

## Results

The percentages of women using four or more ANC, HFD, and PNC were 17.3%, 13.2%, and 10.4% in 2005, respectively, all of which increased slightly to 22.1%, 16.8%, and 13.1% in 2011, respectively (Table 1). In 2005, a high percentage of women (82.3%) had favorable attitudes toward wife beating, and this percentage decreased to 73.7% in 2011. Similarly, the percentage of women with low decision-making power decreased from 32.4% in 2005 to 28.3% in 2011. Table 1 also presents the socioeconomic and demographic characteristics of the sampled women. Notably, several demographic and socioeconomic characteristics had significantly changed from 2005 to 2011. However, among maternal healthcare service utilization only ANC was statistically significant difference from 2005 to 2011.

**Table 1:** Descriptive Statistics of the Study Sample

	2005 (*N* = 6058) %	2011 (*N* = 7043) %
Individual-level characteristics		
Attitudes toward wife beating		
Favorable	82.3	73.7*
Opposing	17.7	26.3
Women’s decision-making power at home		
Low	32.4	28.3*
High	67.6	71.7
Age (years)		
15–24	26.8	25.6
25–34	47.1	49.3
35–49	26.0	25.1
Age at the first marriage (years)		
<16	49.4	42.2*
16–19	33.9	38.6
≥20	16.7	19.2
Religion		
Orthodox	41.1	33.1*
Protestant	18.1	19.3
Muslim	38.2	44.6
Others	3.6	3.0
Educational level		
No education	76.1	67.5*
Primary	15.6	26.2
Secondary and higher	8.3	6.3
Employment status		
No	77.1	70.1*
Yes	22.9	29.9
Child was wanted at the time of the pregnancy		
Yes	69.6	75.4*
No	30.4	24.6
Birth order		
First	17.1	17.6
Second	16.0	16.8
Third or higher	66.9	65.6
Number of children in the household		
1 or 2	38.3	39.2
3 or 4	31.0	30.5
≥ 5	30.6	30.9
Spouse’s educational level		
No education	58.3	50.3*
Primary	26.0	37.7
Secondary and higher	15.6	12.0
Media exposure		
No	88.2	67.9*
Yes	11.8	32.1
Wealth index		
Poorest	24.6	29.8
Poor	18.5	17.4
Middle	18.3	16.3
Rich	17.1	15.8
Richest	21.5	20.6
Place of residence		
Urban	14.7	18.6
Rural	85.3	81.4
Outcome variables		
Antenatal care		
<4 visits	82.7	77.9*
≥4 visits	17.3	22.1
Delivery place		
Home	86.8	83.2
Health facility	13.4	16.6
Postnatal care		
No	89.6	86.9
Yes	10.4	13.1

* *P* < 0.05. Significant difference between years

Table 2 presents the percentages of women using four or more ANC, HFD, and PNC services according to individual-level variables. For 2011 data, of the women with favorable attitudes toward wife beating, 16.4%, 10.3%, and 9.2% had used four or more ANC, HFD, and PNC services, respectively, whereas 38.1%, 32.1%, and 24.0% of the women with opposing attitudes toward wife beating had used four or more ANC, HFD, and PNC services, respectively. In addition, women’s decision-making power was significantly associated with maternal healthcare service utilization. Almost all other bivariate relationships were statistically significant, except for the relationships between whether the child was wanted at the time of pregnancy and the outcomes. The pattern of the results was similar in the 2005 data set.

**Table 2:** Bivariate Relationships of Individual-Level Characteristics with Maternal Healthcare Service Utilization in 2011

	2005 (*N* = 6057)			2011 (*N* = 7043)		
	Antenatal care (%)	Health facility delivery (%)	Postnatal care (%)	Antenatal care (%)	Health facility delivery (%)	Postnatal care (%)
Individual-level characteristics						
Attitudes toward wife beating						
Favorable	13.4*	9.5*	7.6*	16.4*	10.3*	9.2*
Opposing	35.0	30.1	23.1	38.1	32.1	24.0
Women’s decision-making power						
Low	10.3*	7.6*	6.0*	12.5*	9.0*	7.9*
High	20.4	15.8	12.5	25.9	18.8	15.2
Age (years)						
15–24	19.7*	15.6*	11.6*	22.1*	19.4*	14.9*
25–34	18.3	13.8	11.3	23.3	17.2	13.7
35–49	12.8	9.5	7.5	19.7	10.2	10.0
Age at the first marriage (years)						
<16	12.8*	8.7*	6.4*	16.8*	10.5*	9.8*
16–19	18.1	13.0	11.7	22.8	15.3	12.7
≥20	28.9	26.7	19.6	32.4	29.6	21.2
Religion						
Orthodox	23.1*	16.9*	14.4*	31.6*	23.3*	19.1*
Protestant	15.9	11.4	9.3	18.3	11.3	10.6
Muslim	12.6	9.4	7.1	17.4	13.1	4.7
Others	7.8	17.5	4.6	12.1	10.3	2.9
Educational level						
No education	9.4*	6.0*	4.4*	13.1*	7.3*	6.6*
Primary	24.8	17.6	13.6	32.5	23.9	18.0
Secondary and higher	75.0	70.2	59.2	75.7	79.7	62.9
Employment status						
No	15.3*	11.1*	9.0*	18.7*	13.4*	11.7*
Yes	23.8	19.9	15.0	30.2	22.2	16.4
Child was wanted						
No	18.7	13.7	10.8	22.5	16.5	13.3
Yes	16.7	12.9	10.2	22.0	15.9	13.0
Birth order						
First	28.8*	27.6*	19.6*	35.3*	35.1*	25.5*
Second	25.8	15.6	16.2	30.1	24.2	17.4
Third or higher	12.3	8.2	6.7	16.5	8.9	8.7
Number of children in the household						
1 or 2	24.5*	21.2*	16.7*	30.9*	27.7*	20.2*
3 or 4	14.0	10.2	7.9	18.2	11.0	9.9
≥5	10.3	6.1	5.0	14.7	6.1	7.2
Spouse’s educational level						
No education	7.7*	4.7*	3.2*	11.2*	5.6*	5.9*
Primary	17.1	10.8	9.3	26.3	17.4	12.9
Secondary and higher	53.5	48.7	39.2	54.9	56.1	44.1
Media exposure						
No	11.2*	7.3*	5.7*	12.1*	6.4*	6.0*
Yes	60.7	55.3	44.1	43.3	36.5	28.2
Wealth Index						
Poorest	4.4*	4.2*	2.2*	7.5*	5.0*	4.1*
Poor	5.7	4.0	2.4	11.8	4.0	5.1
Middle	9.1	3.3	3.4	15.5	4.5	5.1
Rich	12.5	6.6	5.1	21.8	8.3	10.3
Richest	52.7	44.9	37.0	57.4	57.7	41.3
Place of residence						
Urban	65.0*	59.9*	49.0	58.1*	62.6*	44.2*
Rural	9.1	5.1	3.7	13.9	5.6	6.0

*P < 0.05

Table 3 shows the mean of the community-level characteristics, which were measured by the proportions of women who received primary and higher education, were employed, had opposing attitudes toward wife beating, had high decision-making power, and used contraception In general, the level of all community-level characteristics increased from 2005 to 2011. For instance, the percentage of women with opposing attitudes toward wife beating, high decision-making power, and at least primary education in communities increased respectively from 22.3%, 70.8%, and 36.3% in 2005 to 32.1%, 75.3%, and 48.5% in 2011. All community-level characteristics were positively correlated with each other (*r* = 0.28_0.63).

**Table 3:** Correlation Matrix of Community-Level Variables

	Mean (standard deviation)	A	B	C	D	E	F
2005 (*N* = 535)							
A. Percentage of women with opposing attitudes toward wife beating	22.3(24.0)	1.00					
B. Percentage of women with high decision-making power	70.8(22.9)	0.28*	1.00				
C. Percentage of women with a primary or higher educational level	36.3(29.9)	0.54*	0.38*	1.00			
D. Percentage of women who were employed	28.1(21.4)	0.31*	0.30*	0.40*	1.00		
E. Percentage of women who used contraception	11.3(11.5)	0.36*	0.42*	0.63*	0.36*	1.00	
2011 (*N* = 596)							
A. Percentage of women with opposing attitudes toward wife beating	32.1(27.4)	1.00					
B. Percentage of women with high decision-making power	75.3(22.2)	0.41*	1.00				
C. Percentage of women with a primary or higher educational level	48.5(28.5)	0.60*	0.45*	1.00			
D. Percentage of women who were employed	35.6(19.7)	0.38*	0.29*	0.54*	1.00		
E. Percentage of women who used contraception	17.7(14.6)	0.32*	0.35*	0.51*	0.39*	1.00	

*P < 0.05

The results of the multivariate analyses are presented in Table 4. We first constructed regression models to analyze the interaction effects of year and women’s autonomy on healthcare service utilization and found significant interaction effects (Additional file 1: Table S1). Therefore, we decided to conducted all of our multivariate analyses separately by year. According to the 2005 data, among individual-level variables, opposing attitudes toward wife beating (AOR = 1.47; 95% CI = 1.19–1.81), a higher educational level (AOR = 1.91; 95% CI = 1.35–2.79), a lower number of children in the household (AOR = 0.63, 95% CI = 0.40–0.98) a higher educational level of the spouse (AOR = 1.67; 95% CI = 1.23–2.25), higher media exposure (AOR = 1.36; 95% CI = 1.01–1.83), and a higher wealth index (AOR = 3.58; 95% CI = 1.25–4.75) were associated with a higher likelihood of four or more ANC service utilization.

**Table 4:** Effects of Individual-Level and Community-Level Characteristics on Maternal Healthcare Service Utilization

	2005			2011		
	Antenatal care	Place of delivery	Postnatal care	Antenatal care	Place of delivery	Postnatal care
	AOR(95% CI)	AOR(95% CI)	AOR(95% CI)	AOR(95% CI)	AOR(95% CI)	AOR(95% CI)
Individual-level characteristics						
Attitudes toward wife beating						
Favorable	1.00	1.00	1.00	1.00	1.00	1.00
Opposing	1.47(1.19–1.81)*	1.27(0.99–1.63)	1.23 (0.95–1.58)	1.20(1.01–1.43)*	1.07(0.86–1.35)	1.02(0.81–1.30)
Women’s decision-making power						
Low	1.00	1.00	1.00	1.00	1.00	1.00
High	1.02(0.82–1.26)	1.06(0.83–1.36)	1.04(0.73–1.27)	1.10(0.91–1.32)	1.09(0.88–1.35)	1.04(0.76–1.22)
Age (years)						
15–24	1.00	1.00	1.00	1.00	1.00	1.00
25–34	1.14(0.88–1.48)	1.24(0.89–1.74)	1.27(0.92–1.76)	1.61(1.29–2.03)*	*1.36(1.05–1.77*)*	1.20(0.92–1.55)
35–49	1.11(0.76–1.62)	1.36(0.86–2.14)	1.43(0.90–2.26)	2.29(1.70–3.06)*	1.57(1.06–2.33)*	*1.46(1.04–2.05*)*
Age at the first marriage (years)						
<16	1.00	1.00	1.00	1.00	1.00	1.00
16–19	1.02(0.81–1.28)	1.17(0.75–1.82)	1.22(0.95–1.58)	1.16(0.98–1.37)	0.93(0.75–1.17)	0.89(0.74–1.08)
≥20	1.05(0.78–1.41)	0.82(0.59–1.14)	1.02(0.75–1.39)	0.96(0.76–1.21)	1.15(0.86–1.53)	0.75(0.59–1.01)
Religion						
Orthodox Christianity	1.00	1.00	1.00	1.00	1.00	1.00
Protestant Christianity	0.96(0.67–1.36)	1.21(0.79–1.86)	1.08(0.74–1.58)	0.75(0.56–1.01)	0.90(0.59–1.36)	0.99(0.72–1.40
Islam	1.06(0.78–1.42)	1.38(0.93–2.05)	1.06(0.80–1.40)	1.02(0.82–1.26)	1.28(0.94–1.75)	1.00(0.79–1.30)
Others	0.64(0.34–1.23)	4.48(0.97–6.67)	0.80(0.36–1.81)	0.70(0.41–1.18)	1.45(0.63–3.34)	0.31(0.10–1.80)
Educational level						
None	1.00	1.00	1.00	1.00	1.00	1.00
Primary	1.10(0.87–1.37)	1.03(0.78–1.36)	1.03(0.78–1.37)	1.23(1.03–1.47)*	1.11(0.87–1.41)	1.19(0.96–1.50)
Secondary and higher	1.91(1.35–2.79)*	*1.49(1.05–2.10*)*	*1.88(1.33–2.65*)*	2.03(1.44–2.88)*	2.45(1.65–3.64)*	2.64(1.91–3.65)*
Employment status						
No	1.00	1.00	1.00	1.00	1.00	1.00
Yes	1.14(0.91–1.42)	1.34(1.02–1.76)*	1.20(0.93–1.54)	*1.19 (1.02–1.38*)*	1.08(0.88–1.32)	0.91(0.75–1.10)
Birth order						
First	1.00	1.00	1.00	1.00	1.00	1.00
Second	0.87(0.67–1.21)	0.38(0.28–0.53)*	0.73(0.51–1.03)	0.85(0.67–1.07)	0.51(0.38–0.66)*	0.64(0.48–1.86)
Third or higher	0.82(0.52–1.30)	*0.45(0.26–0.80*)*	1.09(0.64–1.83)	0.68(0.48–0.96)*	*0.54(0.32–0.91*)*	0.76(0.46–1.26)
Number of children in the household						
1–2	1.00	1.00	1.00	1.00	1.00	1.00
3–4	0.69(0.45–1.07)	0.72(0.45–1.13)	0.49(0.31–0.78)*	0.81(0.59–1.12)	0.55(0.34–0.88)*	0.77(0.48–1.22)
≥5	*0.63(0.40–0.98*)*	0.46(0.28–0.74)*	0.50(0.32–0.80)*	0.72(0.52–1.01)	0.58(0.36–0.94)*	0.72(0.44–1.17)
Spouse’s educational level						
None	1.00	1.00	1.00	1.00	1.00	1.00
Primary	1.10(0.89–1.38)	1.13(0.83–1.53)	1.40(1.02–1.91)*	1.36(1.13–1.62)*	1.39(1.06–1.82)*	1.06(0.85–1.33)
Secondary and higher	1.67 (1.23–2.25)*	2.03(1.44–2.86)*	1.86 (1.29–2.68)*	1.66 (1.29–2.16)*	2.24 (1.65–3.05)*	1.88 (1.42–2.50)*
Media exposure						
No	1.00	1.00	1.00	1.00	1.00	1.00
Yes	1.36(1.01–1.83)*	1.46(1.07–2.00)*	1.15(0.85–1.56)	1.65(1.39–1.96)*	1.44(1.13–1.84)*	1.48(1.20–1.82)
Wealth index						
Poorest	1.00	1.00	1.00	1.00	1.00	1.00
Poor	1.03(0.70–1.53)	0.86(0.48–1.57)	0.84(0.48–1.46)	1.15(0.90–1.48)	0.67(0.47–1.00)	1.12(0.80–1.58)
Middle	1.63(1.10–2.42)*	0.68(0.38–1.19)	1.05(0.64–1.84)	1.45(1.13–1.86)*	0.67(0.45–1.01)	1.05(0.72–1.52)
Rich	1.96 (1.31–2.93)*	1.14(0.65–1.99)	1.20(0.69–2.08)	1.59(1.20–2.10)*	0.80(0.51–1.25)	1.66(1.20–2.30)*
Richest	3.58(1.25–4.75)*	2.52(1.34–3.72)*	2.21(1.23–3.97)*	2.24(1.56–3.22)*	1.75 (1.10–2.78)*	2.22(1.46–3.37)*
Place of residence						
Urban	1.00	1.00	1.00	1.00	1.00	1.00
Rural	1.10(0.67–1.79)	0.42(0.23–0.77)*	0.40 (0.23–0.70)*	1.42(0.95–2.12)	0.39(0.25–0.59)*	*0.41(0.28–0.59*)*
Community-level characteristics						
Attitudes toward wife beating^a^	1.16(1.08–1.90)*	1.17(1.13–1.89)*	1.00(0.99–1.01)	1.21(1.01–1.62)*	1.23(1.14–1.96)*	1.18(1.01–1.23)*
Decision-making power^b^	1.64(0.99–1.86)	1.02(0.98–1.07)	1.03(0.99–0.08)	1.14(1.03–1.98)*	1.00(0.98–1.00)	1.01(0.99–1.01)
Educational level^c^	1.38(1.10–1.97)*	*1.31(1.05–1.79*)*	1.18 (1.12–1.27)*	1.15(1.34–1.89)*	1.01(1.00–1.02)*	1.13(1.05–1.96)*
Employment^d^	1.03(0.97–1.05)	1.07(0.99–1.02)	1.03(0.99–1.08)	1.03(0.99–1.10)	1.16(1.04–1.57)*	1.09(0.99–1.01)
Contraception use^e^	1.01(0.98–1.02)	1.01(0.96–1.06)	0.99(0.98–1.01)	1.06(0.99–1.01)	1.04(0.99–1.02)	1.01(0.99–1.08)
Null model						
ICC	0.42*	0.53*	0.49*	0.56*	0.77*	0.54*
Log likelihood	−2941.91	−1973.31	−2169.41	−4607.16	−2644.95	−3437.95
AIC	4207.89	3158.65	3205.10	6051.5	4089.28	4631.05
Final model						
ICC	0.29*	0.40*	0.13*	0.21*	0.33*	0.15*
Log likelihood	−2851.82	−1830.34	−2257.77	−4487.48	−2528.63	−3484.26
AIC	3490.96	2414.95	2537.03	5215.25	3178.29	3954.64

italic * *P*-value <0.05

^a^Percentage of women with opposing attitudes toward wife beating

^b^Percentage of women with high decision-making power

^c^Percentage of women with a primary or higher educational level

^d^Percentage of women who were employed

^e^Percentage of women who used contraception

OR, odds ratio; CI, confidence interval; ICC, intraclass correlation coefficient; AIC, Akaike information criterion

Significant community-level characteristics associated with four or more ANC services were attitudes toward wife beating and educational level. Women in a community with a higher proportion of women opposing wife beating (AOR = 1.16; 95% CI = 1.08–1.90) and having a primary or higher educational level (AOR = 1.31; 95% CI = 1.10–1.97) were more likely to use four or more ANC services. Compared with the predictors in 2005, women’s age, employment status, and decision-making power at the community-level were positively associated with four or more ANC service utilization in 2011.

For HFD service utilization in 2005, a higher educational level, employment status, being first in the birth order, a lower number of children in the household, a higher educational level of the spouse, higher media exposure, a higher wealth index, and urban residency were significant factors positively associated with HFD utilization. For the community-level variables, women in a community with a higher level of opposing attitudes toward domestic violence and a higher level of education were 1.17 (95% CI = 1.13–1.89) and 1.31 (95% CI = 1.05–1.79) times more likely to use HFD. In contrast to the regression results for 2005, individual age and community-level female employment were significantly associated with HFD service utilization in 2011.

For PNC service utilization in 2005, individual-level variables associated with a higher level of PNC service utilization included a higher educational level (AOR = 1.88; 95% CI = 1.33–2.64), a higher educational level of the spouse (AOR = 1.86; 95% CI = 1.29–2.68), and a higher wealth index (AOR = 2.21; 95% CI = 1.23–3.97); however, the number of children in the household and rural residency were negatively associated with PNC service utilization (AOR = 0.50; 95% CI = 0.32–0.80; AOR = 0.40 95% CI = 0.23–0.70, respectively). Among community-level variables, the percentage of women with a primary or higher educational level was positively (AOR = 1.18; 95% CI = 1.12–1.27) associated with PNC service utilization. In 2011, the individual-level characteristics of older age and the community-level characteristic of opposing attitudes toward wife beating were significantly associated with PNC service utilization.

Table 4 indicates that the ICCs for the null model were respectively 0.42, 0.53, and 0.49 in 2005 and 0.56, 0.77, and 0.54 in 2011 for four or more ANC, HFD, and PNC. Compared with the null model, the residual ICC in the final model became smaller (respectively 0.29, 0.40, and 0.13 in 2005 and 0.21, 0.33, and 0.15 in 2011 for four or more ANC, HFD and PNC). This indicated that the addition of the individual-level and community-level variables led to a significant reduction in unexplained variance between communities. However, the variation in the outcomes of maternal healthcare service utilization between communities still remained large and significant.

## Discussion

Our multivariate findings showed that opposing attitudes toward wife beating at the individual level was significantly associated with four or more ANC in both data sets. Compared with the impact of individual-level autonomy variables on maternal healthcare service utilization, the effect of community-level attitudes toward wife beating appeared to be consistently significant over time and across different maternal healthcare services. Women living in a community with a higher percentage of women opposing domestic violence were more likely to use maternal healthcare services even after we controlled for their own attitudes toward domestic violence and other individual-level and community-level characteristics. Because the social and family networks in Ethiopia are generally tight knit, the effect of community norms on individual behavior may tend to exceed that of individual attitudes or preferences [38]. Similar results have been reported in other sub-Saharan African countries [19, 39]. A study conducted in four countries, namely Namibia, Kenya, Nepal, and India, revealed that opposing attitudes toward wife beating measured at the community level affected maternal reproductive healthcare service utilization [40]. In 2014, Ebot et al. also reported a strong association of community-level women’s autonomy on healthcare service utilization and indicated that the preventative healthcare service utilization of Ethiopian women with low individual-level autonomy was significantly increased if they lived in a community with a high level of women’s autonomy [41].

Our results show that in addition to community-level women’s autonomy, the community educational level was strongly associated with maternal healthcare service utilization. Women who lived in communities with a high proportion of women with primary or higher educational levels were more likely to use maternal healthcare services. This result is consistent with those of previous studies suggesting that a community with a high concentration of educated women can improve maternal healthcare service utilization [42, 43]. Education is frequently associated with more access to healthcare services and more knowledge about health behaviors and the healthcare system. A higher concentration of educated women may facilitate the dissemination of knowledge to those with lower educational levels through informal social networks.

Our study also demonstrated that the percentage of employed women in a community was positively associated with HFD service utilization. Along with that for education, this result suggests that the community socioeconomic context affects maternal healthcare service utilization by empowering women educationally, economically, and socially [26]. Notably, the percentage of employed women in the community was significantly associated with HFD but not four or more ANC or PNC service utilization, probably for economic reasons. The estimated cost of HFD (approximately US$17–US$56) is much higher than that of ANC and PNC, which can be obtained at little or no cost [44]. The cost remains a significant barrier for disadvantaged and poor rural women in Ethiopia. Women’s employment may also be associated with other social factors. In areas with a high percentage of employed women, the community is likely to be closer to industrial centers and have more job opportunities, an improved overall economic environment, more health resources, and possibly higher acceptance of HFD. All these social factors may contribute to HFD service utilization.

We observed that some individual-level variables were associated with maternal healthcare service utilization. The results have critical policy implications. After we controlled for other variables, urban residency was associated with increased maternal healthcare service utilization, particularly that of HFD and PNC services. This finding could be attributed to fewer health facilities and a lack of well-functioning transportation systems in rural areas [45]. A mother may not be able to wait for several hours to reach a health facility at the time of delivery. Cultural differences between rural and urban areas may also contribute to the differences in maternal healthcare service utilization. In rural Ethiopia, strong cultural and traditional backgrounds can influence beliefs, norms, and values regarding maternal healthcare service utilization, particularly that of HFD. Misconceptions, such as the belief that a woman undergoes stitching (episiotomy) or cesarean section when delivering at a health facility, may have deterred women from delivering at health facilities [44]. In rural areas, traditional birth attendants have long been part of the culture and are trusted. Traditional attendants may also compete with HFD services by advising mothers to deliver at home and be attended by them. The practice of religion may also play a role in this: some believers may consider medical intervention as a sign of a lack of faith and hence may be unwilling to use HFD services [44].

Women’s socioeconomic status is a highly significant indicator of maternal healthcare service utilization. Similar to previous studies, women’s and their husbands’ educational level, employment status, and wealth index were positively associated with maternal healthcare service utilization [46, 47]. Several studies have identified women’s educational level as a key factor determining the level of maternal healthcare service utilization [12, 48, 49]. Women with a higher educational level are more likely to be knowledgeable regarding the benefits of maternal healthcare service utilization and are more empowered to seek healthcare [50]. In rural Ethiopia where many women have received no formal education (illiterate), informal adult educational interventions for basic maternity care, which intend to increase the health knowledge of women who have not attended school, may increase maternal healthcare service utilization.

Here, media exposure was positively associated with maternal healthcare service utilization. To facilitate changes in the behavior of women, media can be an effective channel of information dissemination, can increase awareness of healthcare facilities, and can motivate interpersonal communication regarding the importance of maternal healthcare services [51]. Researchers have highlighted the requirement for designing and broadcasting public health educational programs through different types of media, particularly for socially disadvantaged groups and low-privileged mothers living in rural and remote areas [52]. Broadcasting health information programs through mass media could play a crucial role in improving population health, including maternal and child healthcare service utilization.

### Study limitations and strengths

Our study has some limitations. First, because of the limited number of variables recorded by the EDHS, we did not examine a full array of factors related to maternal healthcare service utilization, particularly supply-side factors, such as healthcare quality. Second, data on ANC, HFD, and PNC were retrospectively collected from the mothers, potentially resulting in recall bias. Third, adapting community measures by aggregating individual responses to the community level may have increased the likelihood of misclassifying individuals into inappropriate administratively defined boundaries (clusters), potentially resulting in information bias. Finally, the cross-sectional design of this study precluded drawing causal inferences between explanatory factors and maternal healthcare service utilization. Despite these limitations, our study was based on the two EDHS data sets with a nationally representative large sample size. We examined factors influencing three types of maternal healthcare service outcomes. This study demonstrates the importance of individual- and community-level characteristics when investigating women’s autonomy and maternal healthcare service utilization.

## Conclusions

Because of the nature of the cross-sectional design of this study, we are not able to draw causal inference between women’s autonomy and maternal healthcare service utilization. However, the positive relationship between women’s autonomy, socioeconomic factors as well as maternal healthcare service utilization may suggest a requirement for implementing programs and policies to increase women’s autonomy in Ethiopia. The government can focus on both individual awareness and community awareness regarding women’s right for maternal service utilization. Other policies such as the establishment of income-generating programs for women and education programs for enhancing their status and power in both the household and community can effectively improve women’s health and healthcare service utilization.

## Additional file

Additional file 1: Table S1. Effects of Individual-Level and Community-Level Characteristics on Maternal Healthcare Service Utilization (2-year pooled data, 2005 and 2011). Description of data: Results of multivariate analyses about the relationships among individual-level characteristics, community-level characteristics, and interaction effects of women’s empowerment and year on maternal healthcare service utilization. (DOCX 16 kb)

## Abbreviations

AIC: Akaike Information Criterion
ANC: Antenatal Care
AOR: Adjusted Odds Ratio
CI: Confidence Interval
EDHS: Ethiopian Demographic Health Survey
HFD: Health Facility Delivery
ICC: Intraclass Correlation Coefficient
PNC: Postnatal Care
SAS: Statistical Analysis Software
WHO: World Health Organization

## References

[1] Zureick-Brown S, Newby H, Chou D, Mizoguchi N, Say L, Suzuki E, et al. Understanding global trends in maternal mortality. Int Perspect Sex Reprod Health. 2013;39(1):32–41. 10.1363/3903213 PMC3886625 23584466

[2] Global Health Observatory (GHO) data 2015. http://www.who.int/gho/maternal_health/mortality/maternal_mortality_text/en/. Accessed 15 May 2016.

[3] WHO, UNICEF, UNFPA and The World Bank estimates. Trends in maternal mortality: 1990 to 2013. http://www.who.int/reproductivehealth/publications/monitoring/maternal-mortality-2015/en/. Accessed 12 May 2016.

[4] Lassi ZS, Majeed A, Rashid S, Yakoob MY, Bhutta ZA (2013). The interconnections between maternal and newborn health–evidence and implications for policy. J Matern Fetal Neonatal Med.

[5] Victora CG, Requejo JH, Barros AJ, Berman P, Bhutta Z, Boerma T (2016). Countdown to 2015: a decade of tracking progress for maternal, newborn, and child survival. Lancet.

[6] Waiswa P, Pariyo G, Kallander K, Akuze J, Namazzi G, Ekirapa-Kiracho E (2015). Effect of the Uganda newborn study on care-seeking and care practices: a cluster-randomised controlled trial. Global Health Act.

[7] Montgomery AL, Ram U, Kumar R, Jha P, Collaborators MDS (2014). Maternal mortality in India: causes and healthcare service use based on a nationally representative survey. PLoS One.

[8] Nguyen K-H, Hoang V-N, Nguyen KT-B (2014). Are empowered women more likely to deliver in facilities? An explorative study using the Nepal demographic and health survey 2011. Matern Child Health J.

[9] Tarekegn SM, Lieberman LS, Giedraitis V (2014). Determinants of maternal health service utilization in Ethiopia: analysis of the 2011 Ethiopian demographic and health survey. BMC Pregnancy Childbirth..

[10] EDHS. Ethiopia demographic and health survey, 2011. http://dhsprogramcom/pubs/pdf/FR255/FR255pdf. 2011. Accessed Jan 2016:

[11] Shiferaw S, Spigt M, Godefrooij M, Melkamu Y, Tekie M (2013). Why do women prefer home births in Ethiopia?. BMC Pregnancy and Childbirth.

[12] Nigatu D, Gebremariam A, Abera M, Setegn T, Deribe K (2014). Factors associated with women’s autonomy regarding maternal and child health care utilization in bale zone: a community based cross-sectional study. BMC Womens Health.

[13] Pradhan B (2003). Measuring empowerment: a methodological approach. Development.

[14] Sen G, Batliwala S (2000). Empowering women for reproductive rights. POPLINE.

[15] Burra N, Deshmukh-Ranadive J, Murthy RK. Micro-credit, poverty and empowerment: Linking the triad. New Delhi: Sage; 2005.

[16] Upadhyay UD, Karasek D. Women's empowerment and ideal family size: an examination of DHS empowerment measures in sub-Saharan Africa. Int Perspect Sex Reprod Health. 2012;38:78–89. 10.1363/3807812 22832148

[17] Haque SE, Rahman M, Mostofa MG, Zahan MS (2012). Reproductive health care utilization among young mothers in Bangladesh: does autonomy matter?. Womens Health Issues.

[18] Viens LJ, Clouston S, Messina CR (2016). Women’s autonomy and cervical cancer screening in the Lesotho demographic and health survey 2009. Soc Sci Med.

[19] Adjiwanou V, LeGrand T (2014). Gender inequality and the use of maternal healthcare services in rural sub-Saharan Africa. Health Place.

[20] Ononokpono DN, Odimegwu CO (2014). Determinants of maternal health care utilization in Nigeria: a multilevel approach. Pan Afr Med J.

[21] Finlayson K, Downe S (2013). Why do women not use antenatal services in low-and middle-income countries? A meta-synthesis of qualitative studies. PLoS Med.

[22] Heath R (2014). Women’s access to labor market opportunities, control of household resources, and domestic violence: evidence from Bangladesh. World Dev.

[23] Adhikari R (2016). Effect of Women’s autonomy on maternal health service utilization in Nepal: a cross sectional study. BMC Womens Health.

[24] Fotso J-C, Ezeh AC, Essendi H (2009). Maternal health in resource-poor urban settings: how does women’s autonomy influence the utilization of obstetric care services?. Reprod Health.

[25] Kabakyenga JK, Östergren P-O, Turyakira E, Pettersson KO (2012). Influence of birth preparedness, decision-making on location of birth and assistance by skilled birth attendants among women in south-western Uganda. PLoS One.

[26] Osorio AM, Tovar LM, Rathmann K (2014). Individual and local level factors and antenatal care use in Colombia: a multilevel analysis. Cad Saude Publica.

[27] Sado L, Spaho A, Hotchkiss DR (2014). The influence of women's empowerment on maternal health care utilization: evidence from Albania. Soc Sci Med.

[28] Shamaki MA, Buang A (2014). Sociocultural practices in maternal health among women in a less developed economy: an overview of Sokoto state, Nigeria. GMJSS.

[29] Osorio A, Bolance C, Madise N (2015). Community socioeconomic context and its influence on intermediary determinants of child health: evidence from Colombia. J Biosoc Sci.

[30] Asres A, Davey G (2015). Factors associated with safe delivery service utilization among women in Sheka zone, Southwest Ethiopia. Matern Child Health J.

[31] Bayu H, Adefris M, Amano A, Abuhay M (2015). Pregnant women’s preference and factors associated with institutional delivery service utilization in Debra Markos town, north West Ethiopia: a community based follow up study. BMC Pregnancy Childbirth..

[32] Yebyo H, Alemayehu M, Kahsay A (2015). Why do women deliver at home? Multilevel modeling of Ethiopian National Demographic and health survey data. PLoS One.

[33] World Health Statistics 2009. http://wwwwhoint/gho/publications/world_health_statistics/EN_WHS09_Fullpdf. 2009. Accessed 21 Feb 2016.

[34] Ugbor IK, David-Wayas OM, Arua M, Nwanosike DU (2017). The socioeconomic factors that determine women utilization of healthcare Services in Nigeria. Int. j. Asian. soc sci.

[35] Howe LD, Galobardes B, Matijasevich A, Gordon D, Johnston D, Onwujekwe O, et al. Measuring socio-economic position for epidemiological studies in low-and middle-income countries: a methods of measurement in epidemiology paper. Int J Epidemiol. 2012;41:dys037–13. 10.1093/ije/dys037 PMC3396323 22438428

[36] Zamawe CO, Banda M, Dube AN (2016). The impact of a community driven mass media campaign on the utilisation of maternal health care services in rural Malawi. BMC Pregnancy Childbirth..

[37] Snijders TAB, Bosker RJ: Multilevel analysis: an introduction to basic and advanced multilevel modeling, Second Edition. Newbury Park, CA: Sage Publication; 2012.

[38] Jennings EA, Barber JS (2013). The influence of neighbors’ family size preference on progression to high parity births in rural Nepal. Stud Fam Plan.

[39] Fawole OI, Adeoye IA (2015). Women’s status within the household as a determinant of maternal health care use in Nigeria. Afr Health Sci.

[40] Namasivayam A, Osuorah DC, Syed R, Antai D (2012). The role of gender inequities in women’s access to reproductive health care: a population-level study of Namibia, Kenya, Nepal, and India. Int J Womens Health.

[41] Ebot JO (2014). Place matters: community level effects of Women's autonomy on Ethiopian Children's immunization status. Etude Popul Afr.

[42] Mehta SR, Parmar GB, Gamit CL, Mansuri BM, Patel PB, Patel SS (2014). Does maternal education affect maternal and child health care utilization? A community based study in a urban slum area of western India. Int J Interdiscip Multidiscip Stud.

[43] Mekonnen ZA, Lerebo WT, Gebrehiwot TG, Abadura SA (2015). Multilevel analysis of individual and community level factors associated with institutional delivery in Ethiopia. BMC Res Notes..

[44] Roro MA, Hassen EM, Lemma AM, Gebreyesus SH, Afework MF (2014). Why do women not deliver in health facilities: a qualitative study of the community perspectives in south central Ethiopia?. BMC Res Notes.

[45] Chaya N. Poor access to health Services: Ways Ethiopia is overcoming it: Population Action International (PAI). 2007;2(2):1–6.

[46] Akanbi MA, Azuh DE, Adekola PO, Olawole-Isaac A, EJIEGBU CJ (2015). Socio-economic factors influencing the utilization of maternal health care services in Amuwo-Odofin local government area of Lagos state, Nigeria. BEST: IJHAMS.

[47] Mahapatro S (2012). Utilization of maternal and child health care services in India: does women’s autonomy matter. J Fam Welf.

[48] Vora KS, Koblinsky SA, Koblinsky MA (2015). Predictors of maternal health services utilization by poor, rural women: a comparative study in Indian states of Gujarat and Tamil Nadu. J Health Popul Nutr.

[49] Wang W, Hong R (2015). Levels and determinants of continuum of care for maternal and newborn health in Cambodia-evidence from a population-based survey. BMC Pregnancy Childbirth.

[50] Shahabuddin A, Delvaux T, Abouchadi S, Sarker M, De Brouwere V. Utilization of maternal health services among adolescent women in Bangladesh: a scoping review of the literature. Tropical Med Int Health 2015;20(7):822-9. 10.1111/tmi.12503 25757880

[51] Tsawe M, Moto A, Netshivhera T, Ralesego L, Nyathi C, Susuman AS (2015). Factors influencing the use of maternal healthcare services and childhood immunization in Swaziland. Int J Equity Health.

[52] Rai AK, Chauhan BG. Inequality in utilization of maternal health care services among teenage married women in Uttar Pradesh: evidences form NFHS-3. Glob J Multidiscip Stud. 2014;3(10):71–89.

