# Supplementary material for: Women’s autonomy and maternal healthcare service utilization in Ethiopia
Source: BMC Health Serv Res. 2017 Nov 13;17:718. doi: 10.1186/s12913-017-2670-9 (PMC5683361; doi:10.1186/s12913-017-2670-9)
Supplement: Additional file 1: Table S1. — Effects of Individual-Level and Community-Level Characteristics on Maternal Healthcare Service Utilization (2-year pooled data, 2005 and 2011). Description of data: Results of multivariate analyses about the relationships among individual-level characteristics, community-level characteristics, and interaction effects of women’s empowerment and year on maternal healthcare service utilization. (DOCX 16 kb) [file 12913_2017_2670_MOESM1_ESM.docx]

Supplementary Table 1. Effects of Individual-Level and Community-Level Characteristics on Maternal Healthcare Service Utilization (2-year pooled data, 2005 and 2011)

| Characteristics | Antenatal care | Place of delivery | Postnatal care |
| --- | --- | --- | --- |
|  | AOR(95% CI) | AOR(95% CI) | AOR(95% CI) |
| Individual-level characteristics |  |  |  |
| Attitudes toward wife beating |  |  |  |
| Favorable | 1.00 | 1.00 | 1.00 |
| Opposing | 1.05(1.02-1.07)* | 1.04(1.02-1.05)* | 1.00(0.99-1.01) |
| Decision-making power |  |  |  |
| Low | 1.00 | 1.00 | 1.00 |
| High | 1.01 (0.99-1.03) | 1.00(0.99-1.02) | 1.01(1.01-1.02)* |
| Age (years) |  |  |  |
| 15–24 | 1.00 |  |  |
| 25–34 | 1.05(1.03-1.07)* | 1.04(1.02-1.05)* | 0.96(0.95-0.98)* |
| 35–49 | 1.08(1.05-1.10)* | 1.05(1.03-1.07)* | 0.91(0.89-0.92)* |
| Age at the first marriage (years) |  |  |  |
| <16 | 1.00 | 1.00 | 1.00 |
| 16–19 | 1.01(1.00-1.02) | 0.99(0.98-1.00) | 1.02(1.01-1.02)* |
| ≥20 | 1.00(0.98-1.02) | 1.01(0.99-1.02) | 1.05(1.03-1.06)* |
| Religion |  |  |  |
| Orthodox Christianity | 1.00 | 1.00 | 1.00 |
| Protestant Christianity | 0.96 (0.94-0.98)* | 0.99(0.98-1.01) | 1.00(0.98-1.01) |
| Islam | 0.98 (0.97-0.99)* | 1.00(0.99-1.01) | 1.01(1.00-1.01) |
| Others | 0.95 (0.92-0.98)* | 1.06(1.02-1.09)* | 0.99(0.97-1.00) |
| Educational level |  |  |  |
| None | 1.00 | 1.00 | 1.00 |
| Primary | 1.05(1.03-1.07)* | 1.03(1.01-1.04)* | 1.01(1.00-1.02)* |
| Secondary and higher | 1.20(1.16-1.25)* | 1.21(1.17-1.25)* | 1.12(1.09-1.15)* |
| Employment status |  |  |  |
| No | 1.00 | 1.00 | 1.00 |
| Yes | 1.01(1.01-1.03)* | 1.00(0.991.02) | 1.02(1.01-1.03)* |
| Birth order |  |  |  |
| First | 1.00 | 1.00 | 1.00 |
| Second | 0.99(0.97-1.01) | 0.93(0.91-0.95)* | 0.98(0.96-0.99)* |
| Third or higher | 0.96(0.93-0.99)* | 0.92(0.90-0.94)* | 1.00(0.98-1.02) |
| Number of children in the household |  |  |  |
| 1–2 | 1.00 | 1.00 | 1.00 |
| 3–4 | 0.97(0.95-1.00) | 0.97(0.95-0.99)* | 0.99(0.97-1.00) |
| ≥5 | 0.96(0.93-0.98)* | 0.95(0.93-0.97)* | 1.01(1.00-1.03) |
| Spouse’s educational level |  |  |  |
| None | 1.00 | 1.00 | 1.00 |
| Primary | 1.04(1.02-1.05)* | 1.02(1.01-1.03)* | 1.00(1.00-1.01) |
| Secondary and higher | 1.09(1.06-1.12)* | 1.11(1.08-1.13)* | 1.05(1.03-1.07)* |
| Media exposure |  |  |  |
| No | 1.00 | 1.00 | 1.00 |
| Yes | 1.09(1.07-1.11)* | 1.06(1.05-1.08)* | 1.02(1.01-1.04)* |
| Wealth index |  |  |  |
| Poorest | 1.00 | 1.00 | 1.00 |
| Poor | 1.10(1.07-1.13)* | 1.09(1.07-1.12)* | 1.03(1.01-1.05)* |
| Middle | 1.12(1.09-1.15)* | 1.11(1.08-1.13)* | 1.05(1.03-1.06)* |
| Rich | 1.14(1.11-1.18)* | 1.12(1.10-1.14)* | 1.04(1.03-1.06)* |
| Richest | 1.15(1.12-1.19)* | 1.11(1.08-1.14)* | 1.04(1.02-1.06)* |
| Place of residence |  |  |  |
| Urban | 1.00 | 1.00 | 1.00 |
| Rural | 0.89(0.86-0.92)* | 0.77(0.75-0.80)* | 0.93 |
| Year | 1.01(1.00-1.03)* | 1.00(0.99-1.02) | 1.01(1.00-1.02)* |
| 2005 | 1.00 | 1.00 | 1.00 |
| 2011 | 1.01(1.00-1.03)* | 1.00(0.99-1.02) | 1.01(1.00-1.02)* |
| Community-level characteristics |  |  |  |
| Attitudes toward wife beating | 1.09 (1.05-1.12)* | 1.04(1.03-1.05)* | 1.01(0.99-1.03) |
| Decision-making power | 1.00(0.99-1.01) | 1.02(1.01-1.04)* | 1.00(0.99-1.01) |
| Educational level | 1.03(1.01-1.06)* | 1.02(1.01-1.03) | 1.01(1.00-1.02)* |
| Employment | 1.02(0.99-1.04) | 1.04(1.02-1.06)* | 1.02(1.00-1.03)* |
| Contraception use | 1.02(1.00-1.03)* | 1.01(0.99-1.03)* | 1.01(0.99-1.03) |
| Individual attitude toward wife beating*year | 0.99(0.96-1.02) | 1.01(0.98-1.66) | 0.99(0.97-1.01) |
| Decision-making power*year | 1.02(1.01-1.05)* | 1.01(0.99-1.02) | 1.00(0.98-1.01) |
| Community attitude toward wife beating*year | 1.04(0.99-1.10) | 1.12(1.06-1.16)* | 1.00(0.97-1.04) |
| Community decision making power*year | 1.02(0.99-1.05) | 1.002(0.98-1.02) | 1.01(0.99-1.03) |

*P<0.05
